# Supplementary material for: Effect of ripening time on the content of bioactive peptides and fatty acids profile of Artisanal Coalho cheese
Source: PLoS One. 2024 Jul 8;19(7):e0306552. doi: 10.1371/journal.pone.0306552 (PMC11230568; doi:10.1371/journal.pone.0306552)
Supplement: S1 File — Description of the study variables and observed data: Physicochemical parameters, Fatty acid profile, Activity of water-soluble peptides extracted from fresh Artisanal Coalho Cheese (Time 0) and matured for 30, 45 and 60 days. (DOCX) [file pone.0306552.s001.docx]

**Supplementary Table S1. Description of the study variables.**

| Variable Category | Variable name | Variable Description |
| --- | --- | --- |
| Composition – Raw milk | Fat | Milk fat content (g 100/g) |
|  | Total protein | Milk total protein content (g 100/g) |
|  | Lactose | Milk lactose content (g 100/g) |
|  | SNF | Milk solids-not-fat content (g 100/g) |
|  | TS | Milk total solids content (g 100/g) |
|  | Acidity | Milk acidity determined by titration with Dornic solution (g of lactic acid/100 mL) |
|  | SCC | Somatic Cell Count, determined by flow cytometry expressed as the number of somatic cell per milliliter (SC 100/mL) |
|  | SPC | Standard Plate Count, expressed as the number of colony forming units per milliliter (CFU 100/mL) |
| Physio-chemical – Cheese | TS | Cheese total solids content (%) |
|  | Moisture | Cheese moisture, calculated (%) |
|  | Fat | Cheese fat (%) |
|  | SNF | Cheese solids-not-fat, calculated (%) |
|  | FTS | Cheese fat in total solids, calculated (%) |
|  | Protein | Cheese protein content, calculated (%) |
|  | Ash | Cheese ash content, calculated (%) |
|  | pH | Cheese pH value |
| Fatty acid profile – Cheese | Saturated, monounsaturated and polyunsaturated | Types of fatty acid identified on cheeses (%) |
|  | SCFA | Short Chain Fatty Acids (Group of fatty acids, %) |
|  | MCFA | Medium Chain Fatty Acids (Group of fatty acids, %) |
|  | SFA | Saturated Fatty Acids (Group of fatty acids, %) |
|  | UFA | Unsaturated Fatty Acids (Group of fatty acids, %) |
|  | MFA | Monounsaturated Fatty Acids (Group of fatty acids, %) |
|  | FMO | Fatty Acids of Microbial Origin (Group of fatty acids, %) |
|  | AI | Atherogenicity Index (%) |
|  | TI | Thrombogenicity Index (%) |
|  | N6/N3 | Linoleic acid:α-linolenic acid ratio |
| Bioactive peptides – Cheese | WSP | Water-soluble peptides (mg/L) |
|  | DPPH•+ | Antioxidant activity of water-soluble peptides extracted from cheeses, using 1,1-Diphenyl 2-Picryl-Hydrazil (%) |
|  | ABTS+ | Antioxidant activity of water-soluble peptides extracted from cheeses, using 2,2-Azine-bis-(3-Ethylbenzothiazoline)-6-Sulfonic Acid Radical (%) |
|  | ACE-Inhibition | Angiotensin-Converting Enzyme Inhibitory Activity of water-soluble peptides extracted from cheeses (%) |
|  | Antimicrobial activity | Antimicrobial activity of water-soluble peptides extracted from cheeses (%) |

**Supplementary Table S2. Observed data. Physicochemical parameters of fresh Artisanal *Coalho* Cheese (Time 0) and matured for 30, 45 and 60 days.**

| Id | Time | Total Solids (%) | Moisture (%) | Fat (%) | SNF (%) | FTS (%) | Protein (%) | Ash (%) | pH |
| --- | --- | --- | --- | --- | --- | --- | --- | --- | --- |
| A | **0** | 49.48 | 50.52 | 24.92 | 23.23 | 50.36 | 39.82 | 7.21 | 5.4 |
| B | **0** | 50.51 | 49.49 | 23.33 | 27.18 | 46.19 | 38.81 | 6.37 | 5.3 |
| C | **0** | 49.65 | 50.35 | 23.67 | 25.99 | 47.73 | 38.95 | 6.65 | 5.2 |
| D | **0** | 50.68 | 49.32 | 23.33 | 27.35 | 46.06 | 39.35 | 6.63 | 5.1 |
| A | **30** | 66.79 | 33.21 | 27.33 | 39.79 | 40.94 | 33.7 | 7.6 | 4.87 |
| B | **30** | 63.62 | 36.38 | 27.67 | 36.96 | 42.07 | 34.79 | 7.49 | 4.83 |
| C | **30** | 64.58 | 35.42 | 26.67 | 37.92 | 41.34 | 34.65 | 7.27 | 4.83 |
| D | **30** | 62.64 | 37.36 | 26.16 | 36.47 | 41.77 | 36.36 | 7.17 | 5 |
| A | **45** | 69.12 | 30.88 | 28 | 41.12 | 40.53 | 33.05 | 8.23 | 4.9 |
| B | **45** | 67.92 | 32.08 | 28 | 39.92 | 41.32 | 33.29 | 7.75 | 4.93 |
| C | **45** | 67.65 | 32.53 | 28.66 | 38.98 | 42.23 | 35.18 | 8.16 | 5 |
| D | **45** | 69.13 | 30.87 | 28 | 41.13 | 40.54 | 33.85 | 7.44 | 5.06 |
| A | **60** | 68.79 | 31.21 | 20.5 | 39.96 | 42.89 | 38.21 | 7.9 | 4.8 |
| B | **60** | 71.84 | 28.16 | 29.5 | 42.34 | 41.08 | 36.17 | 8.03 | 4.83 |
| C | **60** | 67.45 | 32.55 | 30 | 37.45 | 44.87 | 39.79 | 8.32 | 4.8 |
| D | **60** | 70.37 | 29.63 | 30.5 | 39.87 | 43.4 | 36.96 | 7.05 | 4.83 |

**Supplementary Table S3. Observed data. Fatty acid profile of fresh Artisanal *Coalho* Cheese (Time 0) and matured for 30, 45 and 60 days. (Part I)**

| Rep | Time | C4 | C6 | C8 | C10 | C12 | C13 | C13_iso | C14 | C14_iso | C14_anti | C14_1 | C15 | C16 | C16_1_cis7 |
| --- | --- | --- | --- | --- | --- | --- | --- | --- | --- | --- | --- | --- | --- | --- | --- |
| 1 | 0 | 2.272133 | 2.260809 | 1.542837 | 3.368667 | 3.80695 | 0.09703 | 0.103344 | 11.70644 | 0.304645 | 0.47306 | 0.923964 | 1.097227 | 32.78837 | 0.374064 |
| 2 | 0 | 2.388839 | 2.425911 | 1.605171 | 3.547795 | 3.997092 | 0.099343 | 0.106193 | 12.11316 | 0.313988 | 0.47219 | 0.926225 | 1.114509 | 33.03868 | 0.36264 |
| 3 | 0 | 2.101652 | 2.251754 | 1.513896 | 3.339896 | 3.816526 | 0.0911 | 0.102603 | 11.75007 | 0.376259 | 0.666151 | 0.978157 | 0.592555 | 32.8986 | 0.354792 |
| 4 | 0 | 2.338583 | 2.386363 | 1.592673 | 3.583503 | 4.06647 | 0.102316 | 0.106015 | 12.16641 | 0.311446 | 0.467609 | 0.94157 | 1.109465 | 33.0657 | 0.356402 |
| 1 | 30 | 2.388474 | 2.410826 | 1.577216 | 3.546561 | 4.068492 | 0.100784 | 0.108581 | 12.41621 | 0.335559 | 0.483669 | 0.964806 | 1.142295 | 33.57719 | 0.360433 |
| 2 | 30 | 2.207189 | 2.276962 | 1.511146 | 3.417524 | 3.965163 | 0.098925 | 0.105006 | 12.26101 | 0.330138 | 0.481659 | 0.949844 | 1.131536 | 33.42787 | 0.374412 |
| 3 | 30 | 1.898038 | 2.308705 | 1.624434 | 3.410255 | 3.770829 | 0.100849 | 0.101594 | 11.74484 | 0.30886 | 0.472557 | 0.918205 | 1.108894 | 33.60381 | 0.375089 |
| 4 | 30 | 2.351035 | 2.361936 | 1.553472 | 3.4749 | 4.00741 | 0.100983 | 0.10798 | 12.30824 | 0.327244 | 0.487975 | 0.948494 | 1.136521 | 33.55903 | 0.364155 |
| 1 | 45 | 2.200765 | 2.31134 | 1.52408 | 3.479103 | 4.00736 | 0.097573 | 0.107012 | 12.26333 | 0.320805 | 0.481615 | 0.956373 | 1.132205 | 33.53947 | 0.37616 |
| 2 | 45 | 1.91582 | 2.22531 | 1.483148 | 3.408705 | 3.978495 | 0.100163 | 0.106674 | 12.22501 | 0.330353 | 0.487971 | 0.956961 | 1.123197 | 33.00271 | 0.365946 |
| 3 | 45 | 1.987453 | 2.396538 | 1.562044 | 3.545721 | 4.04963 | 0.101739 | 0.106606 | 12.25445 | 0.327013 | 0.485274 | 0.958741 | 1.134913 | 33.84036 | 0.362649 |
| 4 | 45 | 2.147777 | 2.285401 | 1.504499 | 3.416802 | 3.952138 | 0.096628 | 0.105705 | 12.10629 | 0.320424 | 0.479692 | 0.947377 | 1.111044 | 32.87349 | 0.363477 |
| 1 | 60 | 2.308509 | 2.368158 | 1.56508 | 3.563701 | 4.084052 | 0.102637 | 0.105421 | 12.34226 | 0.322555 | 0.48605 | 0.97211 | 1.139096 | 33.32619 | 0.367829 |
| 2 | 60 | 1.171438 | 2.082728 | 1.621835 | 3.635536 | 3.928185 | 0.101302 | 0.218935 | 11.83465 | 0.308542 | 0.468349 | 0.931394 | 1.112001 | 33.95957 | 0.355578 |
| 3 | 60 | 2.312079 | 2.397982 | 1.580747 | 3.58399 | 4.096008 | 0.1022 | 0.223239 | 12.38281 | 0.323679 | 0.482189 | 0.971517 | 1.139306 | 33.47167 | 0.364045 |
| 4 | 60 | 2.47272 | 2.458842 | 1.602819 | 3.643988 | 4.153834 | 0.10141 | 0.227255 | 12.43024 | 0.324258 | 0.479147 | 0.975714 | 1.139788 | 33.42977 | 0.360682 |

**Supplementary Table S4. Observed data. Fatty acid profile (Saturated, monounsaturated and polyunsaturated) of fresh Artisanal *Coalho* Cheese (Time 0) and matured for 30, 45 and 60 days. (Part II)**

| Rep | Time | C16_anti | C16_1 | C17 | C18 | C18_1_t9 | VA | Oleic | C18_1_c11 | C182_n6 | C183_n3 | Rumenic | CLA_t10c12 | ARA |
| --- | --- | --- | --- | --- | --- | --- | --- | --- | --- | --- | --- | --- | --- | --- |
| 1 | 0 | 0.198751 | 1.771929 | 0.58483 | 9.343297 | 0.125051 | 1.212651 | 17.07626 | 0.532304 | 1.908886 | 0.40363 | 0.33761 | 0.058513 | 0.435827 |
| 2 | 0 | 0.194873 | 1.740597 | 0.589728 | 9.502984 | 0.058725 | 1.180219 | 17.00099 | 0.524071 | 1.543546 | 0.238395 | 0.332063 | 0.03155 | 0.24677 |
| 3 | 0 | 0.201869 | 1.755924 | 0.581438 | 9.584896 | 0.10067 | 1.079794 | 17.69282 | 0.563914 | 1.708243 | 0.389526 | 0.317693 | 0.011794 | 0.328562 |
| 4 | 0 | 0.198973 | 1.738067 | 0.586613 | 9.39152 | 0.058097 | 1.141446 | 16.91592 | 0.515106 | 1.589996 | 0.362336 | 0.348654 | 0.028271 | 0.222782 |
| 1 | 30 | 0.199669 | 1.772362 | 0.612521 | 9.424124 | 0.275077 | 1.177965 | 16.92027 | 0.515733 | 1.439918 | 0.174304 | 0.33631 | 0.010564 | 0.157413 |
| 2 | 30 | 0.201911 | 1.820889 | 0.598591 | 9.394491 | 0.33297 | 1.196185 | 17.39041 | 0.54856 | 1.501726 | 0.21901 | 0.343143 | 0.016447 | 0.171611 |
| 3 | 30 | 0.194646 | 1.753416 | 0.61255 | 10.00113 | 0.370998 | 1.121571 | 17.48076 | 0.563206 | 1.554468 | 0.251397 | 0.324093 | 0.042227 | 0.191431 |
| 4 | 30 | 0.198052 | 1.772953 | 0.609585 | 9.524783 | 0.32012 | 1.127768 | 17.04829 | 0.527523 | 1.48842 | 0.189372 | 0.328572 | 0.028379 | 0.175005 |
| 1 | 45 | 0.204837 | 1.804486 | 0.598291 | 9.452829 | 0.321993 | 1.141684 | 17.21989 | 0.524924 | 1.510586 | 0.193895 | 0.328931 | 0.031405 | 0.161222 |
| 2 | 45 | 0.21159 | 1.868549 | 0.601516 | 9.495771 | 0.336166 | 1.118896 | 18.08228 | 0.581943 | 1.463666 | 0.199615 | 0.358513 | 0.045914 | 0.208452 |
| 3 | 45 | 0.197162 | 1.746049 | 0.60669 | 9.547012 | 0.289385 | 1.093873 | 17.09967 | 0.5137 | 1.424646 | 0.178956 | 0.31945 | 0.033822 | 0.195208 |
| 4 | 45 | 0.212303 | 1.857144 | 0.588676 | 9.354706 | 0.341425 | 1.090015 | 18.27753 | 0.570507 | 1.628091 | 0.187464 | 0.342524 | 0.091442 | 0.167248 |
| 1 | 60 | 0.200549 | 1.780886 | 0.606285 | 9.427806 | 0.329871 | 1.142932 | 17.06663 | 0.523399 | 1.412269 | 0.194583 | 0.346152 | 0.034767 | 0.177762 |
| 2 | 60 | 0.205531 | 1.812747 | 0.614568 | 10.07394 | 0.363106 | 1.069855 | 17.67764 | 0.547986 | 1.462136 | 0.184025 | 0.300721 | 0.032173 | 0.183347 |
| 3 | 60 | 0.196471 | 1.771165 | 0.598882 | 9.453783 | 0.305662 | 1.094316 | 16.99333 | 0.515657 | 1.370271 | 0.175649 | 0.351066 | 0.045691 | 0.17757 |
| 4 | 60 | 0.201365 | 1.757499 | 0.591343 | 9.423982 | 0.365281 | 1.033807 | 16.76496 | 0.507103 | 1.357269 | 0.179663 | 0.34619 | 0.053455 | 0.177134 |

**Supplementary Table S5. Observed data. Antioxidant activity (DPPH•+ and ABTS+) Angiotensin-Converting Enzyme (ACE) Inhibitory Activity of water-soluble peptides extracted from fresh Artisanal *Coalho* Cheese (Time 0) and matured for 30, 45 and 60 days.**

| Rep | Time | Concentration^a^ | DPPH•+ | ABTS+ | ACE-Inhibition |
| --- | --- | --- | --- | --- | --- |
| 1 | 0 | 5 | 65.29 | 57.43 | 43.96 |
| 1 | 0 | 2.5 | 58.01 | 38.95 | 38.81 |
| 1 | 0 | 1.25 | 51.01 | 28.62 | 30.57 |
| 1 | 0 | 0.625 | 44.84 | 24.24 | 27.43 |
| 1 | 30 | 0.312 | 37.51 | 16.62 | 23.69 |
| 1 | 30 | 5 | 76.80 | 78.62 | 59.49 |
| 1 | 30 | 2.5 | 72.19 | 69.67 | 57.58 |
| 1 | 30 | 1.25 | 67.31 | 56.19 | 54.40 |
| 1 | 45 | 0.625 | 61.76 | 45.95 | 52.80 |
| 1 | 45 | 0.312 | 57.43 | 36.24 | 46.59 |
| 1 | 45 | 5 | 82.69 | 72.86 | 67.38 |
| 1 | 45 | 2.5 | 75.63 | 68.29 | 64.30 |
| 1 | 60 | 1.25 | 68.94 | 47.43 | 61.77 |
| 1 | 60 | 0.625 | 57.73 | 37.38 | 59.16 |
| 1 | 60 | 0.312 | 50.00 | 26.14 | 53.74 |
| 1 | 60 | 5 | 72.47 | 59.29 | 61.06 |

^a^Activities carried out in concentrations of 5, 2.5, 1.25, 0.625 and 0.312 mg/L of water-soluble peptides.

**Supplementary Table S6. Observed data. Antimicrobial activity of water-soluble peptides extracted from fresh Artisanal *Coalho* Cheese (Time 0) and matured for 30, 45 and 60 days.**

| Rep | Time | WSP^a^ | *Listeria monocytogenes* | *Pseudomonas aeruginosa* | *Escherichia coli* | *Salmonela typhimurium* |
| --- | --- | --- | --- | --- | --- | --- |
| 1 | 0 | 5 | 31.16 | 37.691 | - | 36.79 |
| 1 | 0 | 2.5 | 21.74 | 24.320 | - | 30.76 |
| 1 | 0 | 1.25 | 14.90 | 13.780 | - | 22.40 |
| 1 | 0 | 0.625 | 9.51 | 4.507 | - | 15.22 |
| 1 | 0 | 0.312 | 4.91 | 1.713 | - | 8.84 |
| 1 | 30 | 5 | 31.16 | 81.564 | 43.02 | 71.50 |
| 1 | 30 | 2.5 | 21.74 | 50.652 | 26.23 | 48.09 |
| 1 | 30 | 1.25 | 14.90 | 45.065 | 11.42 | 38.87 |
| 1 | 30 | 0.625 | 9.51 | 34.264 | - | 30.34 |
| 1 | 30 | 0.312 | 4.91 | 21.862 | - | 26.14 |
| 1 | 45 | 5 | 53.20 | 88.305 | 77.00 | 77.64 |
| 1 | 45 | 2.5 | 43.91 | 60.447 | 68.08 | 70.53 |
| 1 | 45 | 1.25 | 36.85 | 48.454 | 57.09 | 63.21 |
| 1 | 45 | 0.625 | 30.94 | 40.000 | 46.20 | 56.24 |
| 1 | 45 | 0.312 | 22.74 | 32.849 | 40.52 | 49.10 |
| 1 | 60 | 5 | 89.61 | 98.18 | 91.72 | 92.58 |
| 1 | 60 | 2.5 | 77.17 | 73.78 | 65.06 | 85.92 |
| 1 | 60 | 1.25 | 65.69 | 67.67 | 57.99 | 80.76 |
| 1 | 60 | 0.625 | 60.87 | 56.57 | 27.08 | 72.99 |
| 1 | 60 | 0.312 | 49.47 | 37.06 | 15.61 | 66.64 |

^a^WSP: Water-Soluble Peptides. Activities carried out in concentrations of 5, 2.5, 1.25, 0.625 and 0.312 mg/L.

**Supplementary Fig S1 - Images of the cheeses during the experiment.**

| **Fresh – Day 0** | |
| --- | --- |
| 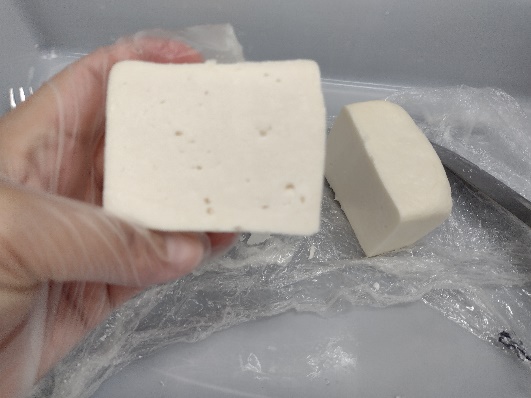  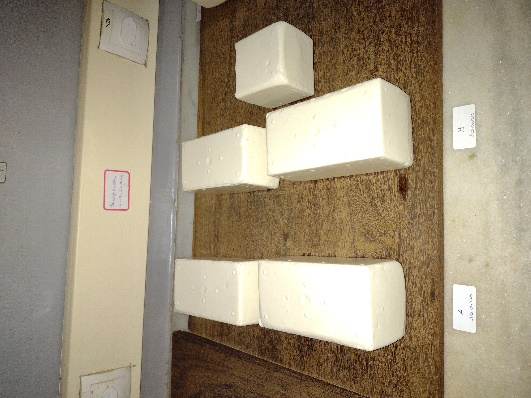 | |
| **Maturated – Day 30** | |
| 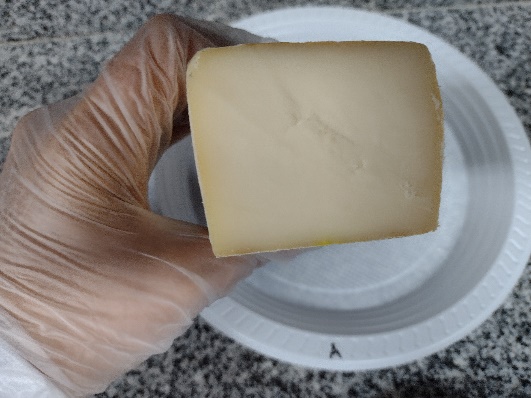 | |
| **Maturated – Day 45** | **Maturated – Day 60** |
| 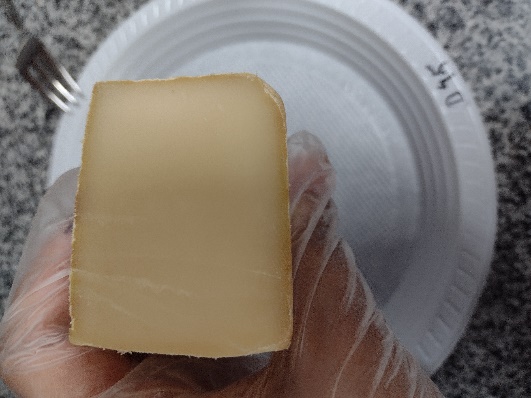 | 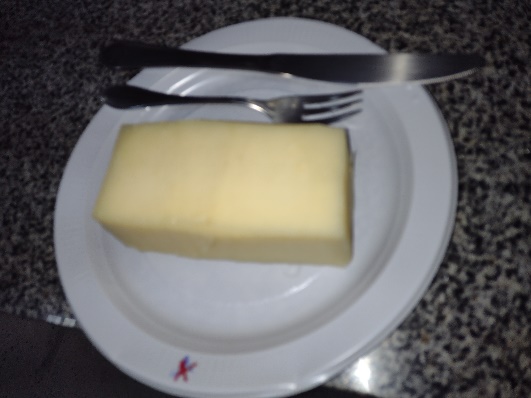 |
